# 1-[1-(3-Methyl­phen­yl)-5-phenyl-4-phenyl­sulfonyl-1*H*-pyrazol-3-yl]ethanone

**DOI:** 10.1107/S1600536811041122

**Published:** 2011-10-12

**Authors:** Mohamed Ghazzali, Hatem A. Abdel-Aziz, Khalid Al-Farhan, Seik Weng Ng

**Affiliations:** a Department of Chemistry, Faculty of Science, King Saud University, PO 2455 Riyadh 11451, Saudi Arabia; b Department of Pharmaceutical Chemistry, Faculty of Pharmacy, King Saud University, PO Box 2457 Riyadh 11451, Saudi Arabia; c Department of Chemistry, University of Malaya, 50603 Kuala Lumpur, Malaysia; d Chemistry Department, Faculty of Science, King Abdulaziz University, PO Box 80203 Jeddah, Saudi Arabia

## Abstract

Both the acetyl and phenyl substituents of the central pyrazole ring in the title compound, C_24_H_20_N_2_O_3_S, are twisted with respect to the pyrazole ring, with the twist involving the phenyl ring being greater [67.4 (1) and 29.6 (2)°]. The tolyl substituent is disordered over two positions in a 1:1 ratio; the mean planes of the aromatic ring are aligned at 67.7 (3) and 69.4 (3)° with respect to the pyrazole ring.

## Related literature

For the synthesis of this class of pyrazoles, which have been tested as anti-inflammatory agents, see: Nasser *et al.* (2011 ▶).
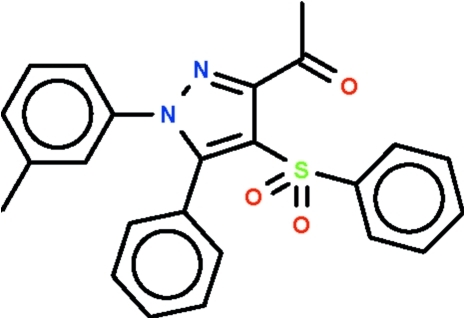


## Experimental

### 

#### Crystal data

C_24_H_20_N_2_O_3_S
*M* *_r_* = 416.48
Monoclinic, 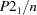

*a* = 10.5717 (4) Å
*b* = 17.7004 (6) Å
*c* = 12.8744 (4) Å
β = 115.945 (1)°
*V* = 2166.30 (13) Å^3^
*Z* = 4
Mo *K*α radiation
μ = 0.18 mm^−1^
*T* = 293 K
0.40 × 0.30 × 0.20 mm

#### Data collection

Rigaku R-AXIS RAPID diffractometer
Absorption correction: multi-scan (*CrystalClear*; Rigaku, 2007 ▶) *T*_min_ = 0.933, *T*_max_ = 0.966
23603 measured reflections
4951 independent reflections
3490 reflections with *I* > 2σ(*I*)
*R*_int_ = 0.042

#### Refinement

*R*[*F* ^2^ > 2σ(*F* ^2^)] = 0.041
*wR*(*F* ^2^) = 0.131
*S* = 1.16
4951 reflections
266 parameters
44 restraints
H-atom parameters constrained
Δρ_max_ = 0.30 e Å^−3^
Δρ_min_ = −0.37 e Å^−3^

### 

Data collection: *CrystalClear* (Rigaku, 2007 ▶); cell refinement: *CrystalClear*; data reduction: *CrystalClear*; program(s) used to solve structure: *SHELXS97* (Sheldrick, 2008 ▶); program(s) used to refine structure: *SHELXL97* (Sheldrick, 2008 ▶); molecular graphics: *X-SEED* (Barbour, 2001 ▶); software used to prepare material for publication: *publCIF* (Westrip, 2010 ▶).

## Supplementary Material

Crystal structure: contains datablock(s) global, I. DOI: 10.1107/S1600536811041122/hg5108sup1.cif

Structure factors: contains datablock(s) I. DOI: 10.1107/S1600536811041122/hg5108Isup2.hkl

Supplementary material file. DOI: 10.1107/S1600536811041122/hg5108Isup3.cml

Additional supplementary materials:  crystallographic information; 3D view; checkCIF report

## Figures and Tables

**Fig. 1.**
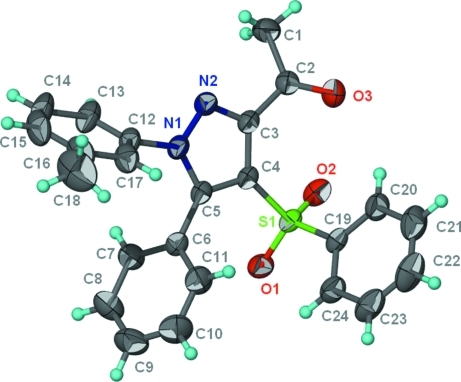
Thermal ellipsoid plot (Barbour, 2001) of the title compound at the 50% probability level; hydrogen atoms are drawn as spheres of arbitrary radius. The disordered tolyl component is not shown.

## supplementary crystallographic information

### Comment

1-[1-(3-Methylphenyl)-5-phenyl-4-(phenylsulfanyl)-1*H*-pyrazol-3-yl]ethanone (Scheme I) exhibited excellent activity compared with a standard drug, indomethacin, when tested as an anti-inflammatory chemical. The high activity has been rationalized by using molecular docking (Nasser *et al.*, 2011). Both the acetyl and phenyl substituents of the central pyrazole ring are twisted with respect to the pyrazole ring, with the twist involving the phenyl ring being greater (67.4 (1) ° and 29.6 (2) °). The tolyl substituent is disordered over two positions in a 1:1 ratio; the mean planes of the aromatic ring are aligned at 67.7 (3) ° and 69.4 (3) ° (Fig. 1).

### Experimental

We have recently reported the synthesis of the compound (Nasser *et al.*, 2011). Crystals were obtained upon recrystallization from ethanol suitable for X-ray structural analysis was obtained by slow evaporation from ethanolic solution at room temperature.

### Refinement

Carbon-bound H-atoms were placed in calculated positions (C–H 0.93–0.96 Å) and were included in the refinement in the riding model approximation, with *U*(H) set to 1.2–1.5*U*(C).

The tolyl group is disordered over two positions; the occupancy could not be refined, and was assumed to be a 1:1 type of disorder. The benzene rings were refined as rigid hexagons of 1.39 Å sides. The temperature factors of the primed atoms were set to those of the unprimed ones, and all anisotropic temperature factors were restrained to be nearly isotropic. The pair of *N*–C_tolyl_ distances were restrained to within 0.01 Å of each other as were the pair of C_methyl_–C_phenylene_ bonds.

Omitted because of bad agreement were (0 8 0), (-2 18 8) and (-8 10 13).

### Crystal data

**Table:**

C_24_H_20_N_2_O_3_S	*F*(000) = 872
*M**_r_* = 416.48	*D*_x_ = 1.277 Mg m^−^^3^
Monoclinic, *P*2_1_/*n*	Mo *K*α radiation, λ = 0.71073 Å
Hall symbol: -P 2yn	Cell parameters from 725 reflections
*a* = 10.5717 (4) Å	θ = 3.1–27.5°
*b* = 17.7004 (6) Å	µ = 0.18 mm^−^^1^
*c* = 12.8744 (4) Å	*T* = 293 K
β = 115.945 (1)°	Block, yellow
*V* = 2166.30 (13) Å^3^	0.40 × 0.30 × 0.20 mm
*Z* = 4	

### Data collection

**Table:**

Rigaku R-AXIS RAPID diffractometer	4951 independent reflections
Radiation source: fine-focus sealed tube	3490 reflections with *I* > 2σ(*I*)
graphite	*R*_int_ = 0.042
ω scans	θ_max_ = 27.5°, θ_min_ = 3.1°
Absorption correction: multi-scan (*CrystalClear*; Rigaku, 2007)	*h* = −13→13
*T*_min_ = 0.933, *T*_max_ = 0.966	*k* = −22→22
23603 measured reflections	*l* = −16→16

### Refinement

**Table:**

Refinement on *F*^2^	Secondary atom site location: difference Fourier map
Least-squares matrix: full	Hydrogen site location: inferred from neighbouring sites
*R*[*F*^2^ > 2σ(*F*^2^)] = 0.041	H-atom parameters constrained
*wR*(*F*^2^) = 0.131	*w* = 1/[σ^2^(*F*_o_^2^) + (0.0582*P*)^2^ + 0.3317*P*] where *P* = (*F*_o_^2^ + 2*F*_c_^2^)/3
*S* = 1.16	(Δ/σ)_max_ = 0.001
4951 reflections	Δρ_max_ = 0.30 e Å^−^^3^
266 parameters	Δρ_min_ = −0.37 e Å^−^^3^
44 restraints	Extinction correction: *SHELXL97* (Sheldrick, 2008), Fc^*^=kFc[1+0.001xFc^2^λ^3^/sin(2θ)]^-1/4^
Primary atom site location: structure-invariant direct methods	Extinction coefficient: 0.0235 (19)

### Fractional atomic coordinates and isotropic or equivalent isotropic displacement parameters (Å^2^)

**Table:**

	*x*	*y*	*z*	*U*_iso_*/*U*_eq_	Occ. (<1)
S1	0.54751 (5)	0.63143 (2)	0.45045 (4)	0.03997 (16)	
O1	0.40174 (15)	0.64090 (7)	0.42277 (13)	0.0545 (4)	
O2	0.64729 (16)	0.62701 (7)	0.56934 (11)	0.0534 (4)	
O3	0.88191 (16)	0.55753 (8)	0.54515 (13)	0.0637 (4)	
N1	0.50448 (17)	0.45192 (8)	0.26398 (13)	0.0440 (4)	
N2	0.63866 (17)	0.43781 (9)	0.34261 (13)	0.0460 (4)	
C1	0.8879 (2)	0.42371 (13)	0.5538 (2)	0.0624 (6)	
H1A	0.9853	0.4310	0.6057	0.094*	
H1B	0.8804	0.3920	0.4909	0.094*	
H1C	0.8409	0.4001	0.5944	0.094*	
C2	0.8211 (2)	0.49846 (11)	0.50784 (17)	0.0452 (4)	
C3	0.67586 (19)	0.49709 (9)	0.41353 (15)	0.0405 (4)	
C4	0.56201 (19)	0.54928 (9)	0.38003 (14)	0.0382 (4)	
C5	0.45247 (19)	0.51785 (9)	0.28374 (15)	0.0388 (4)	
C6	0.3067 (2)	0.54191 (10)	0.21258 (15)	0.0431 (4)	
C7	0.1966 (2)	0.50136 (12)	0.21653 (18)	0.0551 (5)	
H7	0.2151	0.4584	0.2624	0.066*	
C8	0.0593 (2)	0.52496 (16)	0.1521 (2)	0.0722 (7)	
H8	−0.0146	0.4975	0.1542	0.087*	
C9	0.0319 (3)	0.58849 (17)	0.0853 (2)	0.0803 (7)	
H9	−0.0604	0.6048	0.0437	0.096*	
C10	0.1397 (3)	0.62815 (15)	0.0796 (2)	0.0822 (8)	
H10	0.1204	0.6708	0.0331	0.099*	
C11	0.2772 (3)	0.60493 (13)	0.14280 (19)	0.0631 (6)	
H11	0.3501	0.6319	0.1383	0.076*	
C12	0.4517 (7)	0.3942 (4)	0.1774 (6)	0.0362 (12)	0.50
C13	0.4027 (10)	0.3246 (6)	0.1945 (5)	0.0663 (7)	0.50
H13	0.4246	0.3081	0.2690	0.080*	0.50
C14	0.3208 (9)	0.2798 (4)	0.1002 (8)	0.0720 (15)	0.50
H14	0.2880	0.2333	0.1116	0.086*	0.50
C15	0.2879 (7)	0.3045 (4)	−0.0112 (6)	0.0686 (16)	0.50
H15	0.2331	0.2745	−0.0742	0.082*	0.50
C16	0.3369 (10)	0.3741 (6)	−0.0283 (5)	0.0685 (10)	0.50
C17	0.4188 (8)	0.4189 (4)	0.0660 (8)	0.0459 (12)	0.50
H17	0.4516	0.4654	0.0545	0.055*	0.50
C18	0.2992 (15)	0.3922 (5)	−0.1542 (5)	0.1145 (12)	0.50
H18A	0.2297	0.4315	−0.1807	0.172*	0.50
H18B	0.2623	0.3478	−0.2005	0.172*	0.50
H18C	0.3819	0.4087	−0.1608	0.172*	0.50
C12'	0.4183 (7)	0.4036 (4)	0.1680 (6)	0.0362 (12)	0.50
C13'	0.4065 (10)	0.3301 (6)	0.2006 (4)	0.0663 (7)	0.50
H13'	0.4309	0.3190	0.2776	0.080*	0.50
C14'	0.3584 (9)	0.2731 (4)	0.1180 (8)	0.0720 (15)	0.50
H14'	0.3505	0.2239	0.1398	0.086*	0.50
C15'	0.3219 (8)	0.2897 (4)	0.0029 (6)	0.0686 (16)	0.50
H15'	0.2897	0.2516	−0.0524	0.082*	0.50
C16'	0.3337 (10)	0.3633 (6)	−0.0297 (4)	0.0685 (10)	0.50
C17'	0.3819 (8)	0.4203 (4)	0.0529 (8)	0.0459 (12)	0.50
H17'	0.3898	0.4695	0.0312	0.055*	0.50
C18'	0.2985 (14)	0.3918 (2)	−0.1529 (2)	0.1145 (12)	0.50
H18D	0.2052	0.3758	−0.2049	0.172*	0.50
H18E	0.3648	0.3712	−0.1776	0.172*	0.50
H18F	0.3034	0.4459	−0.1527	0.172*	0.50
C19	0.59640 (16)	0.70713 (9)	0.38558 (12)	0.0420 (4)	
C20	0.73709 (16)	0.72002 (10)	0.41591 (12)	0.0567 (5)	
H20	0.8059	0.6889	0.4690	0.068*	
C21	0.7737 (3)	0.78024 (14)	0.3657 (2)	0.0777 (7)	
H21	0.8679	0.7898	0.3853	0.093*	
C22	0.6707 (4)	0.82612 (15)	0.2866 (2)	0.0850 (9)	
H22	0.6961	0.8661	0.2527	0.102*	
C23	0.5320 (3)	0.81318 (13)	0.2580 (2)	0.0755 (7)	
H23	0.4634	0.8446	0.2054	0.091*	
C24	0.4932 (2)	0.75312 (11)	0.30731 (17)	0.0562 (5)	
H24	0.3989	0.7440	0.2878	0.067*	

### Atomic displacement parameters (Å^2^)

**Table:**

	*U*^11^	*U*^22^	*U*^33^	*U*^12^	*U*^13^	*U*^23^
S1	0.0528 (3)	0.0324 (2)	0.0414 (3)	0.00075 (18)	0.0268 (2)	−0.00099 (17)
O1	0.0550 (9)	0.0472 (8)	0.0739 (10)	0.0037 (6)	0.0399 (8)	−0.0035 (7)
O2	0.0773 (10)	0.0465 (8)	0.0364 (7)	0.0045 (7)	0.0249 (7)	−0.0011 (5)
O3	0.0572 (10)	0.0547 (9)	0.0710 (10)	−0.0084 (7)	0.0205 (8)	−0.0132 (7)
N1	0.0500 (10)	0.0366 (8)	0.0474 (8)	−0.0036 (6)	0.0232 (8)	−0.0090 (6)
N2	0.0470 (9)	0.0415 (9)	0.0519 (9)	−0.0001 (7)	0.0239 (8)	−0.0062 (7)
C1	0.0521 (13)	0.0565 (13)	0.0761 (15)	0.0077 (10)	0.0259 (12)	0.0102 (11)
C2	0.0464 (11)	0.0466 (11)	0.0486 (10)	−0.0017 (8)	0.0264 (9)	−0.0043 (8)
C3	0.0470 (11)	0.0337 (9)	0.0464 (10)	−0.0019 (7)	0.0256 (9)	−0.0020 (7)
C4	0.0474 (10)	0.0319 (9)	0.0406 (9)	−0.0021 (7)	0.0242 (8)	−0.0010 (7)
C5	0.0473 (11)	0.0333 (9)	0.0408 (9)	−0.0027 (7)	0.0238 (8)	−0.0008 (7)
C6	0.0495 (11)	0.0381 (10)	0.0404 (9)	−0.0004 (8)	0.0184 (9)	−0.0012 (7)
C7	0.0516 (13)	0.0572 (13)	0.0562 (12)	0.0001 (9)	0.0234 (11)	0.0081 (9)
C8	0.0483 (14)	0.0915 (18)	0.0703 (15)	0.0003 (12)	0.0199 (12)	0.0094 (13)
C9	0.0616 (17)	0.091 (2)	0.0703 (16)	0.0187 (14)	0.0123 (13)	0.0107 (14)
C10	0.084 (2)	0.0708 (17)	0.0720 (16)	0.0151 (14)	0.0159 (15)	0.0280 (13)
C11	0.0649 (15)	0.0553 (13)	0.0619 (13)	−0.0018 (11)	0.0211 (12)	0.0165 (10)
C12	0.022 (3)	0.0355 (19)	0.0547 (14)	0.007 (2)	0.0206 (17)	−0.0108 (14)
C13	0.0873 (18)	0.0454 (16)	0.0844 (16)	−0.0160 (12)	0.0545 (15)	−0.0206 (12)
C14	0.063 (4)	0.0491 (16)	0.117 (3)	−0.016 (2)	0.051 (3)	−0.0338 (17)
C15	0.037 (4)	0.072 (3)	0.092 (2)	0.001 (3)	0.023 (2)	−0.046 (2)
C16	0.0551 (15)	0.085 (3)	0.0636 (14)	−0.0097 (16)	0.0246 (12)	−0.0338 (14)
C17	0.022 (4)	0.0633 (13)	0.049 (2)	−0.0071 (17)	0.012 (2)	−0.0137 (11)
C18	0.109 (3)	0.164 (3)	0.0582 (16)	−0.006 (2)	0.0247 (17)	−0.0346 (18)
C12'	0.022 (3)	0.0355 (19)	0.0547 (14)	0.007 (2)	0.0206 (17)	−0.0108 (14)
C13'	0.0873 (18)	0.0454 (16)	0.0844 (16)	−0.0160 (12)	0.0545 (15)	−0.0206 (12)
C14'	0.063 (4)	0.0491 (16)	0.117 (3)	−0.016 (2)	0.051 (3)	−0.0338 (17)
C15'	0.037 (4)	0.072 (3)	0.092 (2)	0.001 (3)	0.023 (2)	−0.046 (2)
C16'	0.0551 (15)	0.085 (3)	0.0636 (14)	−0.0097 (16)	0.0246 (12)	−0.0338 (14)
C17'	0.022 (4)	0.0633 (13)	0.049 (2)	−0.0071 (17)	0.012 (2)	−0.0137 (11)
C18'	0.109 (3)	0.164 (3)	0.0582 (16)	−0.006 (2)	0.0247 (17)	−0.0346 (18)
C19	0.0583 (12)	0.0319 (9)	0.0389 (9)	−0.0036 (8)	0.0241 (9)	−0.0048 (7)
C20	0.0614 (14)	0.0467 (11)	0.0677 (13)	−0.0072 (9)	0.0333 (11)	−0.0009 (10)
C21	0.0881 (19)	0.0641 (16)	0.0975 (19)	−0.0226 (14)	0.0560 (17)	−0.0040 (14)
C22	0.134 (3)	0.0551 (15)	0.0825 (18)	−0.0239 (16)	0.0628 (19)	0.0065 (13)
C23	0.112 (2)	0.0456 (13)	0.0597 (14)	−0.0017 (13)	0.0294 (15)	0.0126 (10)
C24	0.0689 (14)	0.0422 (11)	0.0514 (11)	0.0000 (9)	0.0208 (11)	0.0041 (9)

### Geometric parameters (Å, °)

**Table:**

S1—O1	1.4316 (14)	C14—H14	0.9300
S1—O2	1.4314 (14)	C15—C16	1.3900
S1—C4	1.7554 (17)	C15—H15	0.9300
S1—C19	1.7713 (15)	C16—C17	1.3900
O3—C2	1.211 (2)	C16—C18	1.525 (6)
N1—N2	1.358 (2)	C17—H17	0.9300
N1—C5	1.361 (2)	C18—H18A	0.9600
N1—C12	1.434 (5)	C18—H18B	0.9600
N1—C12'	1.450 (5)	C18—H18C	0.9600
N2—C3	1.332 (2)	C12'—C13'	1.3900
C1—C2	1.495 (3)	C12'—C17'	1.3900
C1—H1A	0.9600	C13'—C14'	1.3900
C1—H1B	0.9600	C13'—H13'	0.9300
C1—H1C	0.9600	C14'—C15'	1.3900
C2—C3	1.484 (3)	C14'—H14'	0.9300
C3—C4	1.426 (2)	C15'—C16'	1.3900
C4—C5	1.390 (2)	C15'—H15'	0.9300
C5—C6	1.469 (3)	C16'—C17'	1.3900
C6—C11	1.380 (3)	C16'—C18'	1.547 (5)
C6—C7	1.387 (3)	C17'—H17'	0.9300
C7—C8	1.385 (3)	C18'—H18D	0.9600
C7—H7	0.9300	C18'—H18E	0.9600
C8—C9	1.367 (4)	C18'—H18F	0.9600
C8—H8	0.9300	C19—C24	1.381 (2)
C9—C10	1.368 (4)	C19—C20	1.382 (3)
C9—H9	0.9300	C20—C21	1.387 (3)
C10—C11	1.381 (3)	C20—H20	0.9300
C10—H10	0.9300	C21—C22	1.382 (4)
C11—H11	0.9300	C21—H21	0.9300
C12—C13	1.3900	C22—C23	1.366 (4)
C12—C17	1.3900	C22—H22	0.9300
C13—C14	1.3900	C23—C24	1.389 (3)
C13—H13	0.9300	C23—H23	0.9300
C14—C15	1.3900	C24—H24	0.9300
			
O1—S1—O2	118.81 (9)	C13—C14—C15	120.0
O1—S1—C4	107.23 (8)	C13—C14—H14	120.0
O2—S1—C4	108.48 (8)	C15—C14—H14	120.0
O1—S1—C19	107.36 (8)	C16—C15—C14	120.0
O2—S1—C19	108.37 (8)	C16—C15—H15	120.0
C4—S1—C19	105.89 (7)	C14—C15—H15	120.0
N2—N1—C5	113.22 (14)	C15—C16—C17	120.0
N2—N1—C12	111.9 (3)	C15—C16—C18	114.4 (7)
C5—N1—C12	134.9 (3)	C17—C16—C18	125.6 (7)
N2—N1—C12'	126.1 (3)	C16—C17—C12	120.0
C5—N1—C12'	120.7 (3)	C16—C17—H17	120.0
C3—N2—N1	105.36 (14)	C12—C17—H17	120.0
C2—C1—H1A	109.5	C13'—C12'—C17'	120.0
C2—C1—H1B	109.5	C13'—C12'—N1	113.7 (7)
H1A—C1—H1B	109.5	C17'—C12'—N1	124.2 (7)
C2—C1—H1C	109.5	C12'—C13'—C14'	120.0
H1A—C1—H1C	109.5	C12'—C13'—H13'	120.0
H1B—C1—H1C	109.5	C14'—C13'—H13'	120.0
O3—C2—C3	121.22 (18)	C13'—C14'—C15'	120.0
O3—C2—C1	122.0 (2)	C13'—C14'—H14'	120.0
C3—C2—C1	116.76 (17)	C15'—C14'—H14'	120.0
N2—C3—C4	110.24 (16)	C14'—C15'—C16'	120.0
N2—C3—C2	117.59 (16)	C14'—C15'—H15'	120.0
C4—C3—C2	132.17 (16)	C16'—C15'—H15'	120.0
C5—C4—C3	105.91 (15)	C17'—C16'—C15'	120.0
C5—C4—S1	124.43 (14)	C17'—C16'—C18'	113.1 (7)
C3—C4—S1	129.29 (14)	C15'—C16'—C18'	126.9 (7)
N1—C5—C4	105.25 (16)	C16'—C17'—C12'	120.0
N1—C5—C6	122.09 (16)	C16'—C17'—H17'	120.0
C4—C5—C6	132.64 (16)	C12'—C17'—H17'	120.0
C11—C6—C7	119.18 (19)	C16'—C18'—H18D	109.5
C11—C6—C5	121.16 (18)	C16'—C18'—H18E	109.5
C7—C6—C5	119.66 (16)	H18D—C18'—H18E	109.5
C8—C7—C6	119.9 (2)	C16'—C18'—H18F	109.5
C8—C7—H7	120.0	H18D—C18'—H18F	109.5
C6—C7—H7	120.0	H18E—C18'—H18F	109.5
C9—C8—C7	120.2 (2)	C24—C19—C20	121.21 (13)
C9—C8—H8	119.9	C24—C19—S1	119.32 (14)
C7—C8—H8	119.9	C20—C19—S1	119.46 (6)
C8—C9—C10	120.2 (2)	C19—C20—C21	118.73 (15)
C8—C9—H9	119.9	C19—C20—H20	120.6
C10—C9—H9	119.9	C21—C20—H20	120.6
C9—C10—C11	120.2 (2)	C22—C21—C20	120.3 (2)
C9—C10—H10	119.9	C22—C21—H21	119.8
C11—C10—H10	119.9	C20—C21—H21	119.8
C6—C11—C10	120.3 (2)	C23—C22—C21	120.4 (2)
C6—C11—H11	119.9	C23—C22—H22	119.8
C10—C11—H11	119.9	C21—C22—H22	119.8
C13—C12—C17	120.0	C22—C23—C24	120.1 (2)
C13—C12—N1	123.6 (7)	C22—C23—H23	119.9
C17—C12—N1	114.5 (7)	C24—C23—H23	119.9
C14—C13—C12	120.0	C19—C24—C23	119.2 (2)
C14—C13—H13	120.0	C19—C24—H24	120.4
C12—C13—H13	120.0	C23—C24—H24	120.4
			
C5—N1—N2—C3	−1.55 (19)	N2—N1—C12—C17	−116.8 (4)
C12—N1—N2—C3	177.1 (5)	C5—N1—C12—C17	61.4 (7)
C12'—N1—N2—C3	179.6 (5)	C12'—N1—C12—C17	71 (3)
N1—N2—C3—C4	0.88 (19)	C17—C12—C13—C14	0.0
N1—N2—C3—C2	−178.29 (14)	N1—C12—C13—C14	163.3 (5)
O3—C2—C3—N2	150.22 (18)	C12—C13—C14—C15	0.0
C1—C2—C3—N2	−29.7 (2)	C13—C14—C15—C16	0.0
O3—C2—C3—C4	−28.7 (3)	C14—C15—C16—C17	0.0
C1—C2—C3—C4	151.39 (19)	C14—C15—C16—C18	177.0 (10)
N2—C3—C4—C5	0.03 (19)	C15—C16—C17—C12	0.0
C2—C3—C4—C5	179.04 (18)	C18—C16—C17—C12	−176.7 (11)
N2—C3—C4—S1	173.15 (13)	C13—C12—C17—C16	0.0
C2—C3—C4—S1	−7.8 (3)	N1—C12—C17—C16	−164.8 (5)
O1—S1—C4—C5	22.34 (17)	N2—N1—C12'—C13'	54.4 (6)
O2—S1—C4—C5	151.81 (14)	C5—N1—C12'—C13'	−124.5 (3)
C19—S1—C4—C5	−92.06 (15)	C12—N1—C12'—C13'	64 (3)
O1—S1—C4—C3	−149.63 (16)	N2—N1—C12'—C17'	−108.7 (6)
O2—S1—C4—C3	−20.16 (18)	C5—N1—C12'—C17'	72.4 (6)
C19—S1—C4—C3	95.97 (16)	C12—N1—C12'—C17'	−99 (3)
N2—N1—C5—C4	1.57 (19)	C17'—C12'—C13'—C14'	0.0
C12—N1—C5—C4	−176.6 (6)	N1—C12'—C13'—C14'	−163.9 (6)
C12'—N1—C5—C4	−179.5 (5)	C12'—C13'—C14'—C15'	0.0
N2—N1—C5—C6	−176.67 (15)	C13'—C14'—C15'—C16'	0.0
C12—N1—C5—C6	5.1 (7)	C14'—C15'—C16'—C17'	0.0
C12'—N1—C5—C6	2.3 (5)	C14'—C15'—C16'—C18'	179.9 (11)
C3—C4—C5—N1	−0.93 (18)	C15'—C16'—C17'—C12'	0.0
S1—C4—C5—N1	−174.47 (12)	C18'—C16'—C17'—C12'	−179.9 (9)
C3—C4—C5—C6	177.05 (17)	C13'—C12'—C17'—C16'	0.0
S1—C4—C5—C6	3.5 (3)	N1—C12'—C17'—C16'	162.1 (6)
N1—C5—C6—C11	−113.8 (2)	O1—S1—C19—C24	−10.71 (16)
C4—C5—C6—C11	68.5 (3)	O2—S1—C19—C24	−140.20 (14)
N1—C5—C6—C7	67.2 (2)	C4—S1—C19—C24	103.59 (15)
C4—C5—C6—C7	−110.5 (2)	O1—S1—C19—C20	167.98 (11)
C11—C6—C7—C8	−0.9 (3)	O2—S1—C19—C20	38.50 (12)
C5—C6—C7—C8	178.2 (2)	C4—S1—C19—C20	−77.71 (12)
C6—C7—C8—C9	−0.6 (4)	C24—C19—C20—C21	−0.3 (2)
C7—C8—C9—C10	1.6 (4)	S1—C19—C20—C21	−178.99 (16)
C8—C9—C10—C11	−1.1 (4)	C19—C20—C21—C22	−0.2 (3)
C7—C6—C11—C10	1.4 (3)	C20—C21—C22—C23	0.7 (4)
C5—C6—C11—C10	−177.7 (2)	C21—C22—C23—C24	−0.7 (4)
C9—C10—C11—C6	−0.4 (4)	C20—C19—C24—C23	0.3 (3)
N2—N1—C12—C13	79.0 (4)	S1—C19—C24—C23	178.96 (16)
C5—N1—C12—C13	−102.8 (6)	C22—C23—C24—C19	0.2 (3)
C12'—N1—C12—C13	−93 (3)		
